# Adapting to the Destitute Situations: Poverty Cues Lead to Short-Term Choice

**DOI:** 10.1371/journal.pone.0033950

**Published:** 2012-04-18

**Authors:** Lei Liu, Tingyong Feng, Tao Suo, Kang Lee, Hong Li

**Affiliations:** 1 School of Psychology, Southwest University, Chongqing, China; 2 Key Laboratory of Cognition and Personality, Ministry of Education, Chongqing, China; 3 Institute of Child Study, University of Toronto, Toronto, Canada; 4 School of Education, Liaoning Normal University, Dalian, China; Centre national de la recherche scientifique, France

## Abstract

**Background:**

Why do some people live for the present, whereas others save for the future? The evolutionary framework of life history theory predicts that preference for delay of gratification should be influenced by social economic status (SES). However, here we propose that the decision to choose alternatives in immediate and delayed gratification in poverty environments may have a psychological dimension. Specifically, the perception of environmental poverty cues may induce people alike to favor choices with short-term, likely smaller benefit than choices with long-term, greater benefit.

**Methodology/Principal Findings:**

The present study was conducted to explore how poverty and affluence cues affected individuals' intertemporal choices. In our first two experiments, individuals exposed explicitly (Experiment 1) and implicitly (Experiment 2) to poverty pictures (the poverty cue) were induced to prefer immediate gratification compared with those exposed to affluence pictures (the affluence cue). Furthermore, by the manipulation of temporary perceptions of poverty and affluence status using a lucky draw game; individuals in the poverty state were more impulsive in a manner, which made them pursue immediate gratification in intertemporal choices (Experiment 3). Thus, poverty cues can lead to short-term choices.

**Conclusions/Significance:**

Decision makers chose more frequently the sooner-smaller reward over the later-larger reward as they were exposed to the poverty cue. This indicates that it is that just the feeling of poverty influences intertemporal choice – the actual reality of poverty (restricted resources, etc.) is not necessary to get the effect. Furthermore, our findings emphasize that it is a change of the poverty-affluence status, not a trait change, can influence individual preference in intertemporal choice.

## Introduction

The status of poverty and affluence determine many aspects of our social life, from the social activities individuals engaged in [1] to choice preference in social and economic behavior [2]–[3]. It has been shown that ordinary people in poverty countries tend to save less for retirement, eat less healthy food, exercise less regularly than those in wealthy countries [4]. Rational economic theories suggest that ordinary people in the poverty countries have limited resources yet more pressing short-term needs, which prevent them from making long-term investments. Recently, Griskevicius, et al. (2011) [5] showed that mortality cues led individuals who grew up relatively poor to value the present and led individuals who grew up relatively wealthy to value the future. The reasons might be that individuals who grew up relatively poor were associated with fewer resources, greater exposure to threat, whereas individuals who grew up relatively wealthy were associated with more resources and are characterized by economic independence [2], [6]–[7]. In order to adapt to dangerous situations, individuals who grew up relatively poor could need urgently social resources. In addition, previous studies have suggested that subjective social economic status (SES) could be a better predictor of health status than the objective SES 8–9. Disease consequences of feeling poor are often rooted in the psychosocial consequences of being made to feel poor by one's surroundings. Thus, here we propose that the decision to choose sooner-smaller rewards in poverty environment may have a psychological dimension. Poor cues can make people feel that they have fewer resources, greater exposure to threat. Specifically, the perception of environmental poverty cues may induce people alike to favor choices with short-term, likely smaller benefit than choices with long-term, greater benefit.

Although no previous studies have directly tested this hypothesis, existing evidence suggested that psychological factors indeed often affected individuals' intertemporal choices. For example, it has been shown that many individuals chose alternatives with immediate but smaller benefit over delayed but greater reward because the long-term benefits were psychologically discounted [10]–[11]. Further, immediate environmental cues also seem to play an important role. For example, Zhong and DeVoe (2010) [12] found that when participants were exposed to logos of fast food restaurants, they showed more impatience and preferred immediate gains over long-term benefits.

The present study was conducted to explore the impact of cues of poverty on individuals' intertemporal choices. In Experiment 1, we first asked participants to decide initially whether they wished to receive a smaller payment for participation right after the experiment or a larger payment 3 days later. After this pre-test, participants judged photographs in terms of the extent to which they depicted poverty or affluence. After this priming task, participants were asked to choose their preferred manner of subject fee payment, exactly the same as the pre-test. The difference between the post- and pre-test in choices would allow for assessing the effect of poverty cue priming on participants' intertemporal choices. Experiments 2 and 3 were identical to Experiment 1 except for the following: in Experiment 2, instead of the explicit picture judgment task used in Experiment 1, we asked participants to count the number of people shown in the pictures, which served as an implicit priming task; in Experiment 3, participants engaged in a lucky draw game with another partner in which they might draw a prize or nothing, creating an temporary state of “poverty” or “affluence”. Based on the poverty cue hypothesis, we expected that participants would be more inclined to choose the immediate but smaller payment over the delayed but greater payment after being exposed to the “poverty” pictures (resource-deprived environments) or in a state of temporally “poverty” induced by the lucky draw. In contrast, those participants who were exposed to the “affluence” pictures or drew prizes would be more inclined to choose the delayed payment.

## Experiment 1

In Experiment 1, we tested whether explicit exposure to poverty and affluence pictures can induce individuals' different desires to pursue immediate gratification.

### Participants

Two groups of participants were recruited. The first group consisted of 30 college students (*M* = 20.67 years, *SD* = 1.92 years, age range = 18–24 years; 15 females) from a Chinese university. They rated photographs which be used in the priming task. An additional group of 66 college students in Group 2 was recruited from the same university (*M* = 21.70 years, *SD* = 1.91 years, age range  = 18–25 years; 38 females) and they participated in the priming task. All participants gave informed consent prior to their participation in the study. Members of the first group of participants were compensated with a small payment (5 RMB), and members of the second group were compensated with payments based on their performances.

### Materials and Procedures

**Figure 1:**
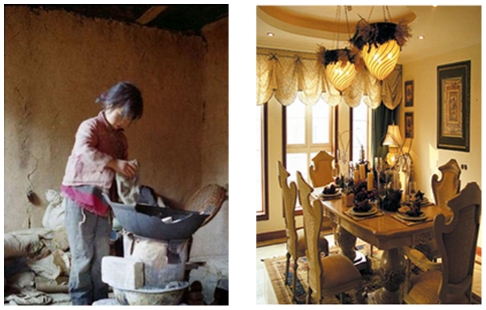
The poverty picture and the affluence picture.

Group 1 rated 44 affluence pictures and 31 poverty pictures as the pretest. In keeping with the previous literature [13], we used a dimensional model for measuring pictures along 3 dimensions, “valence”, “arousal” and “the degree of the poverty-affluence state” and asked an independent sample of 30 participants to rate the respective dimensions on a 9-point Likert scale. So we chose 25 poverty pictures and 25 affluence pictures, in which the disparity of the poverty-affluence state was as large as possible(the poverty picture: *M* = 2.23, *SD* = 1.219; the affluence picture: *M* = 7.76, *SD* = 1.203; *t*(29) = 26.96, *p*<001), and the difference of valence and arousal between poverty pictures and affluence pictures was as small as possible (the poverty picture (valence): *M* = 4.72, *SD* = 1.946; the affluence picture (valence): *M* = 5.97, *SD* = 2.263; *t*(29) = 1.67, *p* = .172; the poverty picture (arousal): *M* = 6.05, *SD* = 2.299; the affluence picture (arousal): *M* = 4.99, *SD* = 2.393); *t*(29) = 1.28, *p* = .233) (see Figure 1 for example).

We tested the prediction that individuals with priming of poverty pictures were more likely to prefer immediate rewards, whereas those with priming of affluence pictures were more likely to choose delayed rewards. For this, participants were assigned randomly to either the “poverty” cue or the “affluence” cue. First, each participant indicated their preference, measured by a standard task, in which each participant made a series of binary choices between ¥*d* today or ¥*d*′ in 3 day (e.g., ¥6 today or ¥9 in 3 day) [8] (pretest). For each trial, the immediate RMB amount (¥*d*) was drawn randomly from a Gaussian distribution with mean ¥6 and standard deviation ¥3, clipped to give a minimum of ¥3 and a maximum of ¥9. The percent difference in renminbi yuan (RMB) amounts between the two rewards ((¥*d*′*−*¥*d*)/¥*d*) was selected from the set {5%, 10%, 15%, 25%, 35%, 50%, 75%, 95%} [14]. Each participant performed 64 trials in a block. Participants were instructed that they would receive one of their choices (randomly selected from the set of all of their choices) at the end of the experiment. Next, they were explicitly told that because of this payment scheme, they should make each choice as though it was the one they were actually going to receive. At the end of the experiment, participants implemented a computer lottery. The procedure could randomly extract a numeral between 1 and 64 in which the numeral indicated the specified trial that determined how much payoff participants got and when participants got their payoff. For example, if participant chose immediate reward in the trial, the money was available at the end of the experiment; if participant chose delayed reward in the trial, the money was available 3 days later. Percentage of immediate rewards (%) chosen in total trials was as the ratio of interest. The higher the ratio of interest, the greater the value participants assigned to immediate rewards. After the choice task, participants in the “poverty” cue completed a task to indicate the extent to which the people in the poverty pictures were poor on a nine-point scale (1-very poor, 9-very affluent; the explicit poverty cue). Participants in the “affluence” cue indicated the extent to which the people in the affluence pictures were affluent on the same nine-point scale (the explicit affluence cue). Then, all of the participants performed another homogeneous intertemporal task (posttest). Finally, they completed the PANAS as a mood measure.

### Results

**Figure 2:**
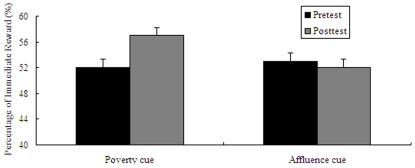
Results from Experiment **1: mean percentage of immediate reward as a function of the poverty and affluence cues (pretest vs. posttest).** Error bars indicate standard errors of the mean.

Two-way ANOVA on the type of pictures (poverty pictures vs affluence pictures) and the type of task (pretest task vs posttest task) revealed that there was a significant interaction between the two factors (*F*(1, 64) = 11.05, *p* = .001). After performing simple effect analysis, the following results were obtained: in the pretest task, there was no significant difference between the preference (Percentage of immediate rewards (%)) in the “poverty” cue (*M* = 53.98%, *SD* = 18.48%) and the preference in the “affluence” cue (*M* = 52.27%, *SD* = 16.73%), *t*(64) = 0.49, *p* = .625. This can be regarded as the homogeneity of participants in the two cues. In the posttest task, compared with participants in the “affluence” cue (*M* = 49.68%, *SD* = 18.76%), participants in the “poverty” cue preferred more instant gratification (*M* = 58.33%, *SD* = 18.93%), *t*(64) = 2.05, *p* = .044. For another simple effect analysis method, participants with the poverty pictures priming preferred more instant gratification in the posttest task (*M* = 58.33%, *SD* = 18.93%) than in the pretest task (*M* = 53.98%, *SD* = 18.48%), *t*(33) = −3.18, *p* = .003. Although participants with the affluence pictures priming preferred more delayed gratification in the posttest task (*M* = 49.68%, *SD* = 18.76%) than in the pretest task (*M* = 52.27%, *SD* = 16.73%), it was not significant, *t*(31) = 1.73, *p* = .093 (see Figure 2). Thus, the disparity of the poverty and affluence cue induced individuals' different desires to pursue immediate gratification.

After the manipulation, participants completed the PANAS as a mood measure. To evaluate whether these effects might have been the result of changes in affective state of the participants, we used mood measure as a covariate in the ANOVA and the result showed that the interaction remained significant (*F*(1, 64) = 9.46, *p* = .003). Thus, our findings indicated that the observed effects were not simply due to difference in affect. In addition, two independent samples T tests were conducted using the Positive Affect and Negative Affect subscales of the PANAS as dependent measures. Results revealed no significant different between the two groups in terms of positive affect (*t*(64) = 0.14, *p* = .889) and negative affect (*t*(64) = −1.46, *p* = .150).

## Experiment 2

In Experiment 2, we examined whether exposure to poverty pictures and affluence pictures, even at an implicit level, can automatically induce individuals' different desires to immediate gratification.

### Participants

A total of 65 undergraduate students from a Chinese university (*M* = 21.72 years, *SD* = 1.94 years, age range  = 18–24 years; 36 females) were assigned randomly to two conditions and the payments of participants were identical to those given in Experiment 1.

### Materials and Procedure

The materials of Experiment 2 were identical to those used in Experiment 1. Participants were first asked to complete an intertemporal choice task (pretest). Then, participants in the “poverty” cue completed an ostensibly unrelated task to count the number of people in the poverty pictures (the implicit poverty cue), while the participants in the “affluence” cue were asked to count the number of people in the affluence pictures (the implicit affluence cue). After the priming task, all of the participants performed another homogeneous intertemporal task (posttest). Finally, they completed the PANAS as a mood measure.

### Results

**Figure 3:**
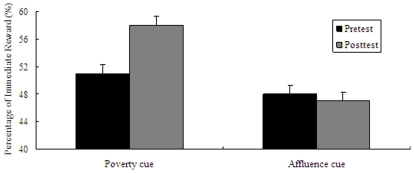
Results from Experiment **2: mean percentage of immediate reward as a function of poverty and affluence cues (pretest vs. posttest).** Error bars indicate standard errors of the mean.

Similar to the result of Experiment 1, two-way ANOVA on the type of pictures (poverty pictures vs affluence pictures) and the type of task (pretest task vs posttest task) revealed that there was a significant interaction between the two factors (*F*(1, 63) = 8.38, *p* = .005). After performing simple effect analysis, the following results were obtained: In the pretest task, there was no significant difference between the preference in the “poverty” cue (*M* = 50.94%, *SD* = 21.32%) and the preference in the “affluence” cue (*M* = 48.22%, *SD* = 16.61%), *t*(63) = 0.58, *p* = .567 (see Figure 3). This can be regarded as the homogeneity of participants in the two cues. In contrast, participants with the poverty pictures priming preferred more immediate gratification in the posttest task (*M* = 58.20%, *SD* = 24.45%) than those with the affluence pictures priming (*M* = 46.94%, *SD* = 19.64%), *t*(63) = 2.06, *p* = .044. For another simple effect analysis method, participants with the poverty pictures priming preferred more immediate gratification in the posttest task (*M* = 58.20%, *SD* = 24.45%) than in the pretest task (*M* = 50.94%, *SD* = 21.32%), *t*(30) = −2.65, *p* = .013. Compared with the pretest task (*M* = 48.22%, *SD* = 16.61%), participants with the affluence pictures priming tended to choose delayed gratification in the posttest task (*M* = 46.94%, *SD* = 19.64%), but it was not significant, *t*(33) = 0.98, *p* = .332 (see Figure 3). Thus, at the implicit level, the exposure to poverty and affluence pictures induced individuals' different preferences in intertemporal choice.

To determine whether the observed effects were the results of changes in mood, we used mood measure as a covariate in the ANOVA and the result showed that the interaction remained significant (*F*(1, 63) = 7.42, *p* = .008). In addition, two independent samples T tests were conducted with the positive and negative affect subscales of the PANAS. Results indicated that the two experimental groups did not significantly differ in their levels of positive affect (*t*(63) = 1.34, *p* = .184) and negative affect (*t*(63) = 0.46, *p* = .646).

## Experiment 3

In Experiment 3, we examined other forms of the poverty and affluence conditions. Here, we created explicit “poverty” and “affluence” status based on wealth in the context of an interactive game. We predicted that the manipulation of the temporary poverty and affluence status can induce individuals' different preferences for immediate gratification.

### Materials and Participants

A total of 62 undergraduate students from a Chinese university (*M* = 21.62 years, *SD* = 1.94 years, age range  = 18–25 years; 36 females) were recruited to participate in the study. They were given payments for their participation.

### Procedure

Each participant received ¥3 basic pay. Then, they indicated their preference in an intertemporal task (pretest). After completing the task, we asked them to play a game [15], in which they were to draw notes labeled “affluence” or “poverty.” The “affluence” (high-pay) participant received another ¥5 bonus to the basic pay (the “affluence” state), whereas the “poverty” (low-pay) participant received no bonus (the “poverty” state). They knew that their counterparts received or did not receive the monetary bonus through the lottery game. Then, all of the participants performed another homogeneous intertemporal task (posttest). Finally, they completed the PANAS as a mood measure.

### Results

**Figure 4:**
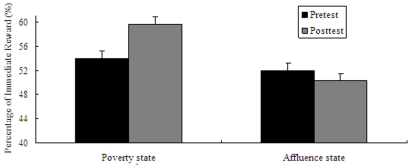
Results from Experiment **3: mean percentage of immediate reward as a function of the manipulations of the “poverty” state and the “affluence” state (pretest vs. posttest).** Error bars indicate standard errors of the mean.

As expected, two-way ANOVA on the type of pictures (poverty pictures vs affluence pictures) and the type of task (pretest task vs posttest task) revealed that there was a significant interaction between the two factors (*F*(1, 60) = 13.03, *p* = .001). After performing simple effect analysis, the following results were obtained: In the pretest task, there was no significant difference between the preference in the “poverty” state (*M* = 53.97%, *SD* = 17.72%) and the preference the “affluence” state (*M* = 52. 01%, *SD* = 16.38%), *t*(60) = 0.45, *p* = .655 (see Figure 4). This can be regarded as the homogeneity of participants. However, participants in the “poverty” state preferred more immediate gratification in the posttest task (*M* = 59.68%, *SD* = 16.15%) than in the “affluence” state (*M* = 50.20%, *SD* = 17.88%), *t*(60) = 2.19, *p* = .032. For another simple effect analysis method, Participants in the “poverty” state preferred more immediate gratification in the posttest task (*M* = 59.68%, *SD* = 16.15%) than in the pretest task (*M* = 53.97%, *SD* = 17.72%), *t*(30) = −3.91, *p*<001. Similarly, although participants in the “affluence” state preferred delayed gratification in the posttest task (*M* = 50.20%, *SD* = 17.88%) relative to the pretest task (*M* = 52. 01%, *SD* = 16.38%), it was not significant, *t*(30) = 1.22, *p* = .232 (see Figure 4). Thus, participants in the “poverty” state seemed impulsive in a manner that could make them prefer to immediate gratification.

As in the previous studies, we examined whether effects of the poverty and affluence status might be caused by changes in mood. We used mood measure as a covariate in the ANOVA and the results showed that the interaction remained significant (*F*(1, 60) = 13.55, *p* = .001). In addition, two independent samples T tests were conducted with the positive and negative affect subscales of the PANAS. Results indicated that the two experimental groups did not significantly differ in their levels of positive affect (*t*(60) = 0.96, *p* = .343) and negative affect (*t*(60) = 0.92, *p* = .360).

## Discussion

Recent studies suggested that the real world decisions (e.g., intertemporal choice) could be influenced by subtle context cues. However, the consequences of the perception of environmental poverty and affluence cues were not adequately understood until now. Based on recent advancements in the behavioral priming literatures, we conjectured that individuals in the poverty cues would likely choose short-term but smaller benefits rather than long-term but greater rewards. In the first two experiments, explicit and implicit exposure to poverty pictures induced individuals to prefer immediate gratification compared with those exposed to affluence pictures. By manipulating the temporal poverty and affluence state, Experiment 3 increased the concern that individuals with perceptions priming of impoverished environments (versus affluent environments) were more likely to choose the immediate but small options. Through the PANAS, we found that overall mood state was not affected by our manipulations.

Why do individuals in poverty cues prefer immediate gratification? Objectively, the poor are associated with fewer resources, greater exposure to threat, and a reduced sense of personal control [2], as opposed to the rich who are associated with more resources and are characterized by economic independence and elevated personal control [6]–[7]. In order to adapt to the dangerous situations, the poor must need more social resources, such as money [16]–[17]. Ultimately, it motivates actions designed to reduce or eliminate the threat and to retain the valuable relationship and the resource. Thus, one prediction from this adaptive view of impulsiveness is that the poor may feel that their poverty state in society prevents them from accessing to the same opportunities as the rich and that the choosing of immediate rewards, in part, derives from a desire to correct for the poverty state [18].

Subjectively, the feeling of poverty may be the core of why the poor prefer immediate gratification. Previous studies have suggested that subjective SES could be a better predictor of health status than the objective SES [8]–[9]. In addition, previous studies have also suggested that disease consequences of feeling poor were often rooted in the psychosocial consequences of being made to feel poverty by the surroundings. As the results of the first two experiments, the manipulation shifted the perception of the participants regarding their relative poverty and affluence status. Thus, both priming (Experiment 1 and Experiment 2) and temporal (Experiment 3) poverty and affluence status may produce the same results: Individuals in poverty state prefer immediate gratification compared with those in affluence state. This indicates that just the feeling of poverty influences intertemporal choice – the actual reality of poverty (restricted resources, etc.) is not necessary to get the effect.

Griskevicius, et al. (2011) [5] showed that, since different life history strategies were shaped by individuals' childhood environments, mortality cues led individuals who grew up in a relatively resource-scarce environment to regard the present as important and led individuals who grew up in a relatively resource-plentiful environment to regard the future as important. Their finding indicated that trait factors (e.g. individuals' childhood environments) influenced economic decisions and risky behaviors. However, many studies have found that social and economic behavior could be influenced by context cues. For example, people who cast their vote within a school were more likely to endorse school funding initiatives on the ballet than others [19]. Similarly, by manipulating participants' temporary perceptions of their social-class rank, lower-class individuals (compared with upper-class individuals) made more accurate inferences about emotion from static images of muscle movements in the eyes [20]. Our results showed that temporary priming of poor cues could actually influence individuals' economic decisions by shifting people life history strategies.

In contrast to the above hypothesis which describes preferences for immediate versus delayed gratification as distinct life history strategies, an equally plausible alternative is that loss/gain frames may play a role in our results. Previous studies showed that loss/gain frames affected risk perception [21], which resulted in choice biases arising from an affect heuristic played by an emotional system [22]. In present research, poor cues which were negative cues were perceived as “loss” frame, while affluent cues which were positive cues were perceived as “gain” frame. Delay exerted its influence on choices via the perceived uncertainty associated with waiting [23]–[24]. The reasons might be that individuals were more sensitive to negative cues (“loss” frame) [25]–[26]. For example, poor cues made individuals more sensitive to their living environments. In other words, poor cues made individuals feel that they had fewer resources, greater exposure to threat. So they chose instant gratification in order to adapt to the dangerous environments [5]. However, our data only demonstrated the idea that environmental cues of poverty vs. affluence could influence intertemporal choice, and it did not speak the process mechanism for our effects. Future work is needed to study the hypothesized process mechanism. Collectively, although previous research showed that life history strategies were shaped and influenced by trait factors (e.g., childhood socioeconomic status) [5], our findings demonstrated that life history strategies were also affected by context factors (e.g., temporary priming concepts of poor).

It is worth noting that while individuals in poverty cues preferred immediate gratification, individuals in affluence cues did not show a significant preference for delayed gratification. Although previous studies showed that the affluence environments could shift preferences toward delayed gratification [5], there are some differences between those studies and our study. For example, our experiments were the priming of temporal poverty and affluence cues (states) (context factor), rather than the change of really poverty and affluence environments (trait factor). From the perspective of loss/gain frames, individuals are less sensitive to positive cues (“gain” frame) than to negative cues (“loss” frame)[25]–[26]. Individuals with the affluent cues priming might not choose delayed gratification, since they were not sensitive to the affluent cues (positive cues).

In Experiment 1 and Experiment 2, we think that participants were made to feel that they were in resource-rich vs. resource-deprived environments, not to feel poor or affluent according to the poverty cue hypothesis. This is in line with our results that poverty cues made individuals instant gratification, and that, in contrast, affluence cues did not make individuals delayed gratification. Since individuals were more sensitive to negative cues than to positive cues [25]–[26], individuals with the affluence cues priming were made to feel less like that they were in resource-rich environment. In addition, our results showed that the affect did not shift as a result of poverty and affluence cues priming, so participants were not made to feel poor or affluent. In Experiment 2, because subjects were asked to focus their attention on another feature (e.g., the number of people) rather than poverty, so we think it is an implicit measure. This experimental method has been shown to be reliably implicit manipulation in previous studies [13], [27].

In our experiment 3, each participant received ¥3 basic pay. Then, we asked them to play a game [15], in which they were to draw notes labeled “affluence” or “poverty.” The “affluence” (high-pay) participant received another ¥5 bonus to the basic pay (the “affluence” state) and the “poverty” (low-pay) participant received no bonus (the “poverty” state). In results, the “poor” have ¥3, and the “rich” have ¥8. Thus, it seems more likely that this is a manipulation of relative poor versus rich, not a manipulation of relative loss versus gain, since loss is considered that participants are taken away from what they have had. This is also in line with previous study which showed that the high-pay participant was considered as the “rich”, and that the low-pay participant was considered as the “poor” [15]. In addition, our findings in Experiment 3 showed that affect did not shift as a result of receiving or not receiving a monetary bonus. We think there are two reasons: on the one hand, each participant first received ¥3 basic pay. On the other hand, after the intertemporal task, participant has another chance to get other bonus from their performance. So the two reasons make participants' affect unchanged.

### Alternative Interpretations

Proponents of evolutionary approaches to human behavior argue that evolutionary analyses can help us understand why humans act as they do because natural selection has shaped the structure of the mental mechanisms that govern human action. Several recent studies have also reported SES disparities in Prefrontal/Executive function. For example, Lipina (2005) reported that infants from lower SES families were, on average, less advanced in the inhibitory control abilities [28]. Studies of adults with neuropsychological tests converge on the same conclusion, showing SES disparities in tests of executive function [29]. The long developmental trajectory of prefrontal regions might be expected to render them particularly susceptible to environmental influence [30]. And, in some situations, preferring small present gains over large future ones can be rational due to adaptations to reproductive success and life history trade-offs. According to this perspective of evolution, decision mechanisms should fit the environment in which they operate–temporal preferences should be ‘ecologically rational’ rather than economically rational [31]. Thus, the poor should discount the future more steeply than the rich in order to adapt to present survival.

By manipulating the relative poverty and affluence status of the participants, our results showed that individuals in the poverty cues preferred immediate gratification and those in the rich cues tended to prefer delayed gratification. This is in line with previous studies which indicated that real world decisions with significant economic consequences could be influenced by subtle context features [12]. Furthermore, our findings emphasize that it is a change of the poverty-affluence status, not a trait change, can influence individuals' preference in intertemporal choice. However, it is possible that the persistent change of the poverty-affluence status in the future may lead to a difference of brain's function.
